# Lay epidemiology and the interpretation of low‐risk drinking guidelines by adults in the United Kingdom

**DOI:** 10.1111/add.13072

**Published:** 2015-08-22

**Authors:** Melanie Lovatt, Douglas Eadie, Petra S. Meier, Jessica Li, Linda Bauld, Gerard Hastings, John Holmes

**Affiliations:** ^1^ School of Health and Related ResearchUniversity of SheffieldSheffieldUK; ^2^ Institute for Social MarketingUniversity of StirlingStirlingUK; ^3^ UK Centre for Tobacco and Alcohol Studies, Nottingham, UK

**Keywords:** Alcohol, drinking practices, drinking guidelines, lay epidemiology, qualitative, units

## Abstract

**Aims:**

To explore how the concept of lay epidemiology can enhance understandings of how drinkers make sense of current UK drinking guidelines.

**Methods:**

Qualitative study using 12 focus groups in four sites in northern England and four sites in central Scotland. Participants were 66 male and female drinkers, aged between 19 and 65 years, of different socio‐economic backgrounds. Data were analysed thematically using a conceptual framework of lay epidemiology.

**Results:**

Current drinking guidelines were perceived as having little relevance to participants' drinking behaviours and were generally disregarded. Daily guidelines were seen as irrelevant by drinkers whose drinking patterns comprised heavy weekend drinking. The amounts given in the guidelines were seen as unrealistic for those motivated to drink for intoxication, and participants measured alcohol intake in numbers of drinks or containers rather than units. Participants reported moderating their drinking, but this was out of a desire to fulfil work and family responsibilities, rather than concerns for their own health. The current Australian and Canadian guidelines were preferred to UK guidelines, as they were seen to address many of the above problems.

**Conclusions:**

Drinking guidelines derived from, and framed within, solely epidemiological paradigms lack relevance for adult drinkers who monitor and moderate their alcohol intake according to their own knowledge and risk perceptions derived primarily from experience. Insights from lay epidemiology into how drinkers regulate and monitor their drinking should be used in the construction of drinking guidelines to enhance their credibility and efficacy.

## Introduction

Low‐risk drinking guidelines are a widely used alcohol policy internationally and enjoy strong support from the alcohol industry and governments as a non‐regulatory intervention which does not directly limit individual freedoms. They are argued to have several functions, which include communicating evidence on risks of drinking to the public to inform consumption choices and providing an objective definition of risky drinking for use in medical practice 1, 2. In its 2012 Alcohol Strategy, the UK Government announced a review of its lower risk drinking guidelines 2. The purpose of this was to take account of new evidence on the health consequences of drinking emerging since 1995, when the guidelines were last reviewed. Drawing on the concept of lay epidemiology, this study aimed to inform the government's review by exploring adult drinkers' interpretations and use of current drinking guidelines within the context of their knowledge and practices around the regulation of their alcohol consumption.

Researchers have questioned the value and effectiveness of drinking guidelines 3, 4, 5, 6, and evidence suggests that they are not perceived as useful by drinkers 7, and that awareness of drinking guidelines does not lead necessarily to reductions in risky drinking 8 or a perception that drinking above the guidelines constitutes risky behaviour 9. A small number of qualitative studies have suggested that guidelines' apparent failure to change drinkers' behaviours or perceptions can be explained partly by their perceived lack of relevance to how alcohol is consumed in everyday social situations, and a failure of public health messaging to acknowledge that pleasure, sociability and intoxication are among the principal motivations for consuming alcohol 4, 9, 10. Findings that awareness of public health guidance does not necessarily result in people engaging in less risky practices have been observed in other areas of public health research 11, 12, 13. Responding to this, researchers have noted that public health guidance privileges epidemiological knowledge and related conceptions of risk while failing to address lay knowledge and risk perceptions 12, 14, 15. This is argued to limit the effectiveness of such guidance 4, 16.

One approach to addressing this deficit is for research and practice to give greater attention to lay epidemiology, which comprises knowledge and beliefs about health and causation of disease which are constructed primarily from subjective experience, observation of family and social networks and media sources 11, 14, 17. This contrasts with (standard) epidemiology which claims an objective understanding of aetiology based on statistical evidence 11, 14. Additionally, while standard epidemiology focuses largely on health outcomes at a population level, lay epidemiology takes a more holistic approach, where perceived health risks are considered within the context of other aspects of individuals' lives and are weighed against perceived benefits and values 11, 14. By emphasizing the individual and social contexts within which people make sense of health and illness, it is argued that lay epidemiology can enhance the relevance and effectiveness of public health guidance 14.

Lay epidemiology fits within a social constructivist ontology which considers social phenomena—in this case illness—not as biological or natural ‘givens’ as in objectivist understandings of the social world, but as concepts that emerge from social interactions, which hold different meanings depending on the cultural and historical contexts in which they are produced and experienced 18, 19. For example, social constructivist approaches have critiqued ways in which obesity has been conceptualized as a ‘biological reality’ as opposed to a social phenomenon, and how this has been used to regulate and order people's bodies 20, 21. Within a constructivist paradigm, there is an emphasis on how people interpret and make sense of social phenomena, and how these interpretations relate to actions, practices and behaviours.

While lay epidemiology has been used to understand failures to comply with public health guidance in contexts such as smoking 13, HIV 12 and coronary heart disease 11, it has yet to be applied explicitly to alcohol. The literature has, instead, focused on drinkers' rationales and motivations for and risk perceptions surrounding drinking, and considered the findings in the context of debates around drinking guidelines 9, 10. Although much of this work relates to issues which are relevant to lay epidemiology, no previous work has sought to analyse drinkers' responses to low‐risk guidelines with an explicitly lay epidemiology focus or given consideration to how those responses might be incorporated into future guidelines. In this study, we explore how drinkers interpret the current UK drinking guidelines in the context of their own drinking practices and risk perceptions. Interpreting the findings within a lay epidemiology framework, we suggest how evidence and insights derived from drinkers' understandings of risks associated with alcohol consumption can be incorporated into the construction of drinking guidelines to enhance their credibility with, and relevance for, their intended audience.

## Methodology

The findings presented in this paper form part of a larger study, APISE (Alcohol Policy Interventions in Scotland and England), which aims to assess the impact and effectiveness of alcohol control policies in each country. Twelve focus groups ranging in size from three to six participants (total *n* = 66) were conducted in England and Scotland (six in each) in February and March 2014. Independent market research consultants identified, recruited door‐to‐door and received informed consent from participants who were purposively sampled to be male and female drinkers (defined as anyone who drinks alcohol at least twice per year) aged 19–65, of lower and higher socio‐economic backgrounds (measured using occupation of the household's highest earner). Each focus group comprised participants of the same gender and similar ages and socio‐economic backgrounds (see Table 1), as we expected participants sharing these characteristics to be more likely to share cultural norms around drinking, and therefore feel comfortable discussing their own drinking practices and beliefs with others. Moderators were careful, however, to encourage discussion of differences of opinion and dissenting narratives, in order to avoid a ‘false consensus’ 22. The focus groups took place in a variety of settings (e.g. community halls, hotels) in eight different locations in northern England and central Scotland). Researchers based at the University of Sheffield (M.L. and J.L.) moderated the English groups and a researcher based at the University of Stirling (D.E.) moderated the Scottish groups. Participants were each given £25 for taking part and to cover travel expenses. Ethical approval was granted by the Universities of Sheffield and Stirling.

**Table 1:** Constitution of focus groups.

*Group no*.^a^	*Age* (*years*)	*Gender*	*Social grade* b	*Number attending*
E1	19–24	F	C2DE	6
E2	19–24	M	ABC1	4
E3	25–44	F	C2DE	6
E4	25–44	M	C2DE	6
E5	45–65	F	ABC1	6
E6	45–65	M	ABC1	6
S1	19–24	F	C2DE	5
S2	19–24	M	ABC1	6
S3	25–44	F	C2DE	6
S4	25–44	M	ABC1	3
S5	45–65	F	ABC1	6
S6	45–65	M	C2DE	6
Total				66

a ‘E' denotes English groups; ‘S' denotes Scottish groups.

b We used a demographic classification, which is standard in the United Kingdom and classifies social grades according to occupation. ABC1 includes professional/skilled workers and C2DE includes unskilled/manual/unemployed.

A semi‐structured topic guide was developed by the lead author in consultation with other members of the research team. The first section focused on participants' awareness and understanding of the concept of drinking guidelines, and explored whether and how participants monitored their own drinking. In the second section we explored participants' responses to current drinking guidelines. Prompt cards displaying the UK guidelines were shown, and prompts displaying the Australian and Canadian drinking guidelines (with quantities converted to UK units; 1 unit = 10 ml/8 g ethanol) were also circulated to explore reactions to alternative guidelines. The drinking guidelines which were shown to the participants are displayed in Table 2.

**Table 2:** UK, Australian and Canadian drinking guidelines.

*Country*	*Regular guideline*	*Single occasion guideline*
United Kingdom	Men/women should not regularly drink more than 3–4/2–3 units a day (regularly means drinking this amount most days or every day)	After an episode of heavy drinking, it is advisable to refrain from drinking for 48 hours to allow tissues to recover [this information is only available in the detailed guidance]
Australia	For healthy men and women, drinking no more than 3 units on any day reduces your risk of harm from alcohol‐related disease or injury over a life‐time	Drinking no more than 5 units on a single occasion reduces the risk of alcohol‐related injury arising from that occasion
Canada	Reduce your long‐term health risks by drinking no more than:	Reduce your risk of injury and harm by drinking no more than 5 units (for women) and 7 units (for men) on any single occasion
	• 17 units a week for women, with no more than 3 units a day most days	Plan to drink in a safe environment. Stay within the weekly limits outlined in guideline 1
	• 25 units a week for men, with no more than 5 units a day most days	
	Plan non‐drinking days every week to avoid developing a habit	

The focus groups were audio‐recorded and transcribed verbatim. The transcripts were analysed inductively using a coding frame which combined the categories in the topic guide with those which emerged from initial reading of the transcripts. The conceptual tool of lay epidemiology also guided our analysis, in that particular attention was paid to how participants referred to their own rationales for moderating their alcohol consumption. Transcripts were coded to the emerging categories using NVivo version 10 software, and the coding framework was checked and revised following discussions between M.L. and D.E. in order to agree that the names given to codes accurately reflected the meaning in the transcripts. Once all transcripts had been coded, categories which contained similar meanings were then grouped into overarching themes 18. We then re‐read the original transcripts in order to check that our interpretations and the overarching themes made sense and could be defended with respect to the transcript data.

## Results

### Guidelines are a poor fit with participants' drinking practices

In general, the drinking guidelines were disregarded by participants and there appear to be three primary interrelated factors which may help to explain this. These are: (1) a disconnect between the guidelines, which are focused on regular drinking, and the participants' tendency to drink irregularly, including occasional binge drinking; (2) a disconnect between the amounts given in the guidelines and participants' typical consumption levels; (3) difficulties measuring and monitoring units. While participants disregarded the guidelines, they referred to other rationales constructed from their own risk perceptions to monitor their drinking, demonstrating how lay epidemiology informed and addressed their perceived risks of alcohol.

#### Poor fit with typical drinking patterns

Participants interpreted the guidelines within the context of their own behaviour and justified disregarding them by referring to their own rationales for moderating their consumption. For instance, participants of all ages felt that having a guideline for daily use was unhelpful, as they did not drink on most days: ‘[b]ecause we don't drink every day you just don't take it in’ (E6 1Participant ID number corresponds to the focus group of which the participant was a member. See Table 1 for more information.). The guidelines were seen as more relevant for ‘people who drink every day probably [people agreeing] more than people who just drink at the weekend’ (E1). As most participants drank only at weekends, a weekly guideline was preferred, because: ‘then you can do it to suit yourself during the week I would say’ (S5). Some felt that as they did not drink during the week, they did not need to monitor their consumption at the weekend:

I probably wouldn't consider it if I was drinking at the weekend and it was my only night of relaxing with a drink during the week, I probably wouldn't care if I was over the limit or not because it wouldn't be greatly and I'd just think that I don't do it often enough (S3).

The perception that the guidelines are targeted at frequent drinkers rather than occasional bingers may have informed the participants' preference for the Australian and Canadian guidelines, which include separate guidelines for regular drinking and for single occasion drinking. This ‘two‐guideline approach’ was seen as more flexible and of more relevance:

I think that's definitely better because you've got your [single occasion] one that says ‘don't go over five if you are going to have a binge’, and you've got if you're just going to have a light drink during the week you've got the three units most days, it kind of suits everybody (S4).

Participants' preferences for guidance on heavy, single‐occasion drinking coheres with their positive responses to a more detailed, but less commonly displayed (e.g. not on product labels), version of the UK guidelines which advises people to leave 48 hours without drinking after a heavy drinking session. However, while participants commented that they did not drink following such occasions, this was due less to health concerns or guidance and more a reflection of practical issues, such as needing to go to work, childcare responsibilities or recovering from hangovers: ‘I wouldn't say you'd pay attention to it because they're saying it, you just pay attention because your body's telling you’ (S2).

#### Guidelines are not relevant to participants' typical consumption levels

The daily UK guidelines were seen as broadly realistic for people who drank every day, but because the majority of participants did not drink regularly throughout the week but drank a great deal at weekends, a recommendation of not regularly drinking more than 2–3 units for a woman or 3–4 units for a man was seen as unrealistic: ‘[t]heir too much is not our too much. According to that, their too much is like ‘I've only just started’ to be honest’ (E4); ‘If I'm having a drink and I'm out, I don't want to stop at three drinks really if I'm having, if I'm at a night out, I'm not going out to get drunk but you know, three drinks is gone, especially three skinny glasses, well that's not even a bottle of wine is it when you're out?’ (S5).

For many participants, one of the main motivations of drinking was to get drunk, and the guidelines were interpreted within that context. Consequently, they were disregarded as they were not seen to acknowledge this motivation. While the single‐occasion guidelines of Australia and Canada have higher ‘limits’, these were still regarded as unrealistically low: ‘like say five units, it don't seem like a lot, that just seems like a normal, like a normal drink, like I'd say five, I'd say binge drinking should be way more than five’ (E6); ‘[i]t's probably not really, like the average adult I wouldn't have thought five units would have made them particularly drunk’ (S3). While some participants acknowledged that exceeding the guidelines might lead to harm, rejection of the guidelines was rationalised by balancing risks against the perceived social benefits of drinking: ‘I think I would have to accept that if you drank more than the guidance that was given there then you could argue that there is a greater chance of harm as a consequence of drinking. But to, I think we'd have to weigh that up against the desire to be sociable and so to some there's a, you know, you're prepared to take that [risk] aren't you? (E2)’; ‘I'm aware that… binge drinking… has some detrimental potential effects, but I, if you like, waiver that in favour of going out and socializing and having a good time’ (E6).

Other participants acknowledged the health risks while continuing to drink above the guidelines, as they felt that ‘it would never happen to them’. Rationales offered by participants to explain this view reflected lay interpretations of key epidemiological concepts such as relative and absolute risk. This included appraising their alcohol intake against others who drank more than them: ‘I'm probably a fool to myself; I never, I just palm it off as if it's never going to happen to me because I don't drink in excess. I always look further down… to people who are drinking every day kind of thing’ (E4). Attitudes towards drinking in relation to long‐term health conditions did not generally appear to be linked strongly to age or gender, although one participant from the 19–24 age group regarded himself as not at risk, as he anticipated drinking less as he got older and associated health harms with drinking heavily over a long period of time:

although I sort of appreciate the long term like risks of drinking a lot I'm not particularly bothered because I just think I'm 21, I'm at university so after, as soon as I finish university it will probably slow down a lot when I have a full‐time job so I don't really think about it in the long term either (E2).

#### Problems of measuring and monitoring units and self‐regulation

While awareness of the use of units in the drinking guidelines was high, participants reported that they thought about their own alcohol consumption in terms of bottles or drinks, rather than units: ‘you just count the drink, you don't count the units or anything’ (S3); ‘I think it would be better if it was like two to three cans or two to three whatever it was, or one bottle of whatever’ (E4). Many participants identified practical barriers to sticking to a limited number of units in both the on and off‐trade:

And there's like a whole culture of buying everyone drinks so that the people who shove like one in my face and it's like yeah, yeah, yeah… no one's thinking ‘oh how many units have I had?’ (E2).

[w]ell that's it as well, especially if you're at home you're not going to have a unit are you, not what a pub would serve you, so their units, [because] they do measure them, would be different to our units at home, so it would be quite different (E5).

Generally, participants regarded measuring alcohol in standard container sizes as more viable, and questioned the usefulness of units in guidelines: ‘I think when people say ‘units’ a lot of people are going to go, “oh I don't know about [those] units”, whereas if you went, “oh you can only have five cans a day” they'd be like, “oh okay, five cans”’ (E4).

Despite generally rejecting units as a way of monitoring their drinking, participants gave clear examples of how they regulated their drinking in other ways, which were relevant to their motivations for drinking and broader social values. Strategies for self‐regulation included not drinking at all, drinking more slowly and limiting the number of drinks consumed. Other participants spoke of ‘knowing their own limits’ and bodies, and sometimes regulated themselves by switching to different types of drink according to how they believed they would feel the next day: ‘I could have vodkas all night and I'd wake up and I might feel a bit ropey but wine makes me feel like I'm like hit by a bus’ (S3); ‘I know when to stop drinking because I… know that if I have another one I'm going to be ill. So you know’ (E6).

However, this regulation was informed more by their commitment to fulfilling valued responsibilities such as childcare and employment and was unrelated to the long‐term health risks which typically inform drinking guidelines: ‘I usually let the wife have a sleep in on a Saturday morning. So I get up early and do all the baby things so I don't want to be getting up feeling awful and with a thick head’ E6); ‘I'm a child minder so I'd be very conscious not to be really drunk obviously and yeah, if I'm going out and driving and you know, I'll just stick to one drink, if that, because I kind of think sometimes it's better to have nothing at all, don't risk it sort of thing’ (S3). Consequently ‘regulation’ did not always mean limiting the amount of alcohol consumed for long‐term health consequences, but limiting the immediate negative effects or consequences of drinking which would be felt the following day.

## Discussion

In general, participants perceived that the guidelines lacked relevance to their drinking practices for three primary reasons. First, daily guidelines were seen as irrelevant by drinkers whose drinking patterns comprised heavy weekend drinking. Secondly, the amounts given in the guidelines were seen as unrealistic for those motivated to drink for intoxication. Thirdly, participants measured alcohol intake in numbers of drinks or containers rather than units. However, despite generally disregarding the guidelines, participants regulated their drinking in ways, and for reasons, which were meaningful to them.

As a holistic approach to understanding health and illness, lay epidemiology assesses health risks in the context of a wide range of additional concerns, including the perceived benefits of risky activities, and individual and societal norms, attitudes and values. For example, participants explained their disregard for guidelines with reference to their motivations for and experiences of drinking, which often focused on pleasure and intoxication and the perceived failure of guidelines to account for this 4, 6, 9, 16. Perceived benefits of drinking were also seen as justifications for disregarding guidelines, echoing previous work which noted drinkers’ views that drinking with others facilitated companionship and sociability, and that drinking generally had a relaxing effect described as a stress‐relief 23. These nuanced interpretations of guidelines contrast with the approach commonly taken by public health professionals which focuses primarily on improving health and wellbeing at a population level and draws heavily upon epidemiological evidence with a more reductionist structure (e.g. relative risks of mortality as a function of average grams of alcohol consumed per day). As an approach to shaping and informing health behaviours, this paradigm typically fails to incorporate the broader concerns above which are embedded in lay epidemiology. Public health guidance can create a ‘disconnect’ for those receiving the guidance as a result 6. The implication of this is that greater account needs to be taken of individual and societal perspectives on drinking alongside epidemiological risk evidence when designing guidelines 14, 24.

While the participants in this study did not generally adhere to the drinking guidelines, they were not impervious to the risks of alcohol consumption and monitored and moderated their drinking in ways that made sense to them. For instance, participants monitored their consumption in numbers of drinks rather than units, a finding which supports claims that units are a flawed metric for people who measure consumption in ways more meaningful to them, which include physiological and emotional experiences 5. It is also notable that the reasons given by participants for moderating their drinking were typically short term and not health‐related. This can be interpreted within a lay epidemiological framing of health, which emphasizes the ability to function and perform valued responsibilities with which excessive drinking would interfere 12. Most participants stated that they either did not drink at all during the week, or drank only small amounts because of their need to work and provide childcare. When drinking, participants spoke of knowing and adhering to their own ‘limits’. Like similar accounts of self‐regulation in previous research, such limits were not based on drinking guidelines but were subjective and experiential, based on how they felt and their predictions of how they would feel the next day 5, 25, 26.

Viewed within a framework of lay epidemiology, which understands that people's health behaviour decisions are made within the context of their knowledge, experience and values, the participants acted rationally 14. An alternative but related interpretation is that the apparent ‘failure’ of people to adhere to public health guidance may be explained in terms of the prevention paradox 14, 27: although adhering to guidelines might result in the improvement of the population's health as a whole, most individuals are unlikely to see a noticeable improvement in their own health, and therefore lack a sufficient incentive to comply with health guidelines. Public health guidance, which acknowledges people's concerns about the impacts which their behaviour has on valued responsibilities and relationships, may be more effective than guidance which relies upon individuals changing their behaviour purely because of health considerations 14. However, there is a tension between designing public health guidance which fits lay epidemiology and that which fits standard epidemiology. Simply accommodating the concerns of the public may lead to important public health problems being inadequately addressed. Therefore, the challenge for those designing guidance is to embed public health concerns within a framing which fits the public's perceptions and motivations. This may be accompanied by efforts to reframe those perceptions and motivations to give greater immediate attention to long‐term health risks.

The disjuncture between the guidelines' concern with long‐term health issues and the participants' primary focus on social problems also reflects long‐standing debates over what sort of a problem alcohol is, whose responsibility it is to solve it, and how this should be done 28, 29. The rationale behind the development of the UK drinking guidelines has been predominantly health‐based. The publication of the UK drinking guidelines of 1987, which advised that men should drink no more than 21 units a week and women no more than 14 units a week, was largely the result of campaigns by health practitioners warning of the associations between alcohol use and the development of long‐term health conditions 28. The revision of the guidelines to daily, rather than weekly, amounts in 1995 was made primarily on evidence which suggested the cardioprotective effects of regularly drinking low levels of alcohol 30. However, during this time there was also a growing discourse among policymakers of broader, non‐health‐specific ‘alcohol‐related harms’ such as crime and acute injury. These ‘competing interpretations of the alcohol problem’ 29 were implied by the publication of the 2012 Alcohol Strategy by the Home Office rather than the Department for Health, and the presentation of minimum unit pricing as a response to anti‐social behaviour and binge drinking rather than as a measure designed to address long‐term health conditions 31. The results presented above suggest that drinking guidelines might have greater impact if they acknowledge both the health and social dimensions of problems associated with alcohol.

To our knowledge, this is the first study in any country to examine qualitatively the interpretation and use of drinking guidelines by the general adult population. While the sample is not representative of the whole UK population, and caution should be exercised in generalizing from the findings, our research drew upon perspectives from a diverse sample of drinkers (*n* = 66) from two parts of the United Kingdom, and given the similar approaches taken to drinking guidelines across high income countries 32, 33, our findings are likely to have wider relevance to international policy makers developing new or revised guidelines. Participants' attitudes did not appear to be rooted strongly in demographic characteristics, with different age, gender and socio‐economic groups expressing similar responses. While exploring these differences was not a focus of our research, the homogeneity of attitudes is surprising, and could be explored in future research.

A number of implications emerge from our findings. First, the disconnect between drinking practices and guidelines may be addressed partly by separate regular and single occasion guidelines as used in Canada and Australia but not currently in the United Kingdom 34. Secondly, the disconnect between guidelines and motivations for and experiences of drinking may be addressed by guidelines referencing the social concerns and cultural values which frame drinkers' behavioural decisions. For example, communicating the long‐term risks of drinking rather than just guideline thresholds 35, and using narrative‐based messages which show the impact which a person's drinking (in the short or long term) can have on family and work life. Thirdly, our paper supports a growing body of research which suggests that the use of units to measure consumption is neither meaningful nor helpful to many drinkers 5, 7, 36. However, our research suggests that drinkers use other strategies and methods for regulating their alcohol consumption, and understanding and harnessing these may be useful in developing future guidance.

## Conclusions

Drinking guidelines derived from, and framed within, solely epidemiological paradigms lack relevance for adult drinkers who monitor and moderate their alcohol intake according to their own knowledge and risk perceptions derived primarily from experience. Insights from lay epidemiology into how drinkers regulate their drinking should be used in the construction of drinking guidelines in order for them to have enhanced credibility and efficacy.

### Declaration of interest

None.
